# Rapid resolution of guttate psoriasis with upadacitinib: a case report

**DOI:** 10.3389/fmed.2026.1899043

**Published:** 2026-07-29

**Authors:** Zenan Tang, Xiaoyang Liu

**Affiliations:** Department of Dermatology, Peking University People's Hospital, Beijing, China

**Keywords:** case report, guttate psoriasis, intractable pruritus, JAK1 inhibition, upadacitinib

## Abstract

We report a 30-year-old woman with acute, severe, and intensely pruritic Guttate Psoriasis (GP) who experienced a rapid and marked response to the oral selective JAK1 inhibitor upadacitinib. The patient presented with a 20-day history of rapidly spreading, intensely pruritic lesions after an upper respiratory infection, with a mildly elevated antistreptolysin O titer but no clinical or laboratory evidence of ongoing active infection. Notably, although she had previously responded well to secukinumab, an IL-17A inhibitor, the current flare occurred just 1 week after a scheduled injection. Conventional therapies, including antihistamines, topical steroids, and narrowband ultraviolet B phototherapy, failed to control either disease progression or the debilitating pruritus. Histopathological examination revealed epidermal hyperkeratosis with parakeratosis, acanthosis, elongation and downward extension of the rete ridges, dilated capillaries in the papillary dermis, and scattered lymphocytic infiltration, supporting the diagnosis of guttate psoriasis. After active infection had been excluded, upadacitinib 15 mg daily was initiated as an off-label rescue strategy. The patient reported significant pruritus relief within the first 24 h. After 2 weeks of treatment, near-complete clearance of skin lesions was achieved, and the patient completed a total of 28 days of upadacitinib therapy, resulting in complete resolution of the eruption. She subsequently underwent tonsillectomy, and no recurrence was observed during 6 months of follow-up. This case suggests that short course upadacitinib may represent a valuable therapeutic option for severely pruritic GP when rapid disease control is needed, but only after active infection has been excluded with careful safety monitoring.

## 1 Introduction

Guttate Psoriasis (GP) is an acute, eruptive form of psoriasis distinguished by the sudden onset of numerous small, scaly, drop like papules, frequently following an upper respiratory tract infection, particularly streptococcal pharyngitis (1). Although GP is often self-limiting and may resolve spontaneously within 3, 4 months, it can lead to significant physical discomfort and psychosocial distress, especially in children and young adults (2). In a subset of patients, GP may recur or progress into chronic plaque psoriasis, necessitating longer term management (3). Streptococcal infection is the best recognized trigger, but the evidence supporting routine antibiotic treatment for established GP remains limited; antibiotics are generally most appropriate when active streptococcal infection is present (4–6). In addition, GP has been reported after viral infections, including coxsackievirus, SARS-CoV-2, and herpes simplex virus, indicating that diverse infectious

stimuli may precipitate psoriatic inflammation in susceptible individuals (7–9). First-line therapeutic strategies for GP typically include supportive care, treatment of proven underlying infections, topical corticosteroids, and narrowband ultraviolet B (NB-UVB) phototherapy (4). Systemic immunosuppressants such as methotrexate or cyclosporine are reserved for severe or refractory cases due to their potential adverse effects and monitoring requirements (10). Biologics, primarily anti-IL-17, anti-IL-23, and anti-IL-12/23 inhibitors, have also been reported to be effective in GP, but current evidence remains limited to small retrospective studies and case reports, and large scale clinical trials are lacking (11–13).

The Janus kinase (JAK)/signal transducer and activator of transcription (STAT) pathway plays a critical role in the signaling of multiple pro-inflammatory cytokines implicated in psoriasis pathogenesis, including IL-23, IL-17-related downstream networks, and interferon-gamma (14, 15). Oral JAK or TYK2 pathway inhibitors, including tofacitinib and deucravacitinib, have demonstrated efficacy in psoriatic disease or plaque psoriasis, supporting the therapeutic relevance of this signaling axis (15–17). Upadacitinib, an oral selective JAK1 inhibitor, has shown robust efficacy in psoriatic arthritis and atopic dermatitis (18, 19). In addition, JAK1-dependent cytokine signaling is closely related to non-histaminergic itch pathways, providing a mechanistic rationale for rapid pruritus relief in highly pruritic inflammatory dermatoses (20, 21). Nevertheless, the application of upadacitinib in acute, severe GP remains largely unexplored. Here, we report a case of a 30-year-old woman with severe, pruritic GP who experienced rapid relief of pruritus within 24 h, a marked cutaneous response to upadacitinib, and near-complete clearance within 2 weeks, suggesting a potential therapeutic avenue for challenging GP cases.

## 2 Case description

A 30-year-old woman presented with a 20-day history of a rapidly spreading, intensely pruritic rash. She had experienced two previous episodes of guttate psoriasis-triggered by respiratory infections, the most recent of which occurred 1 year earlier and had been refractory to conventional therapies but was successfully controlled with secukinumab. Thereafter, she had been receiving regular secukinumab maintenance therapy and had received her scheduled injection 1 week before the onset of the current eruption. Two weeks before symptom onset, she developed an upper respiratory infection.

The eruption initially appeared on the neck and subsequently spread to the trunk, limbs, and palms, presenting as widespread edematous erythematous papules and plaques with prominent scaling (Figure 1). Laboratory evaluation showed a mildly elevated antistreptolysin O titer, while the complete blood count and erythrocyte sedimentation rate were essentially within normal limits; together with the absence of fever, pharyngalgia, tonsillar exudate, lymphadenopathy, or other clinical signs of ongoing infection, these findings did not support active infection at the time of treatment decision making. Despite treatment with antihistamines, topical steroids, a compound traditional Chinese medicine preparation, and narrowband ultraviolet B phototherapy, the eruption continued to progress, and the debilitating itch severely disrupted her sleep. At baseline, the physician global assessment (PGA) score was 3, the pruritus numerical rating scale (NRS) score was 9, and the Dermatology Life Quality Index (DLQI) score was 19, indicating moderate-to-severe disease activity, severe pruritus, and a marked impairment in quality of life. A skin biopsy was therefore performed and confirmed the diagnosis of psoriasis. Histopathological examination revealed epidermal hyperkeratosis with parakeratosis, acanthosis, elongation and downward extension of the rete ridges, dilated capillaries in the papillary dermis, and scattered lymphocytic infiltration, supporting the diagnosis of guttate psoriasis (Figure 2).

**Figure 1:**
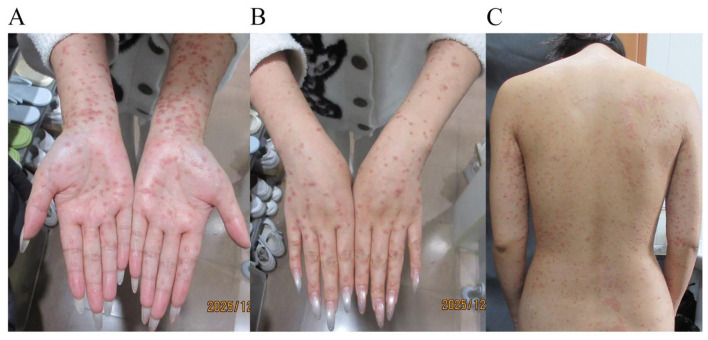
Clinical manifestations before upadacitinib therapy. **(A)** Numerous erythematous, scaly papules and plaques on the bilateral palms and volar forearms. **(B)** Similar erythematous, scaly lesions on the dorsal hands and forearms. **(C)** Widespread erythematous papules and plaques with scaling on the back and upper arms.

**Figure 2:**
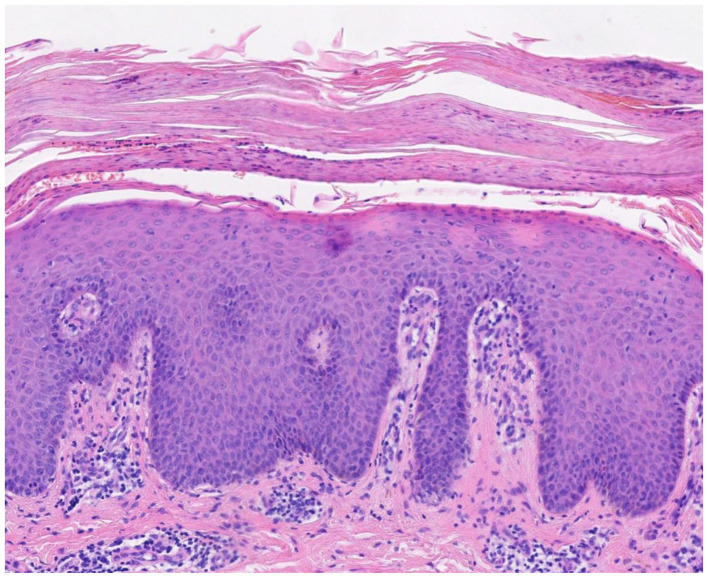
Dermatopathology photomicrographs.

Given the acute and severe nature of the flare, together with profound treatment-resistant pruritus despite ongoing biologic therapy, a switch in therapeutic strategy was considered. After ensuring that no active infection was present and after discussing the off-label nature of treatment and potential risks, oral upadacitinib 15 mg daily was initiated. The response was rapid: the patient reported significant relief from pruritus within the first 24 h. After 1 week of treatment, the pruritus NRS score decreased to 2; at this time, the lesions began to darken, scaling markedly diminished, and the disease stabilized. After 2 weeks, the rash showed further improvement, as shown in (Figure 3); the PGA score improved to 1, the pruritus NRS score decreased to 1, and the DLQI score decreased to 12. The patient completed a total of 28 days of upadacitinib therapy, at which point complete resolution of the acute eruption had been achieved. At week 4, the PGA score was 0, the pruritus NRS score was 0, and the DLQI score had decreased to 4. Upadacitinib was subsequently discontinued. She later underwent tonsillectomy, and no recurrence was observed during 6 months of follow-up. The medication was well tolerated, with no adverse events reported.

**Figure 3:**
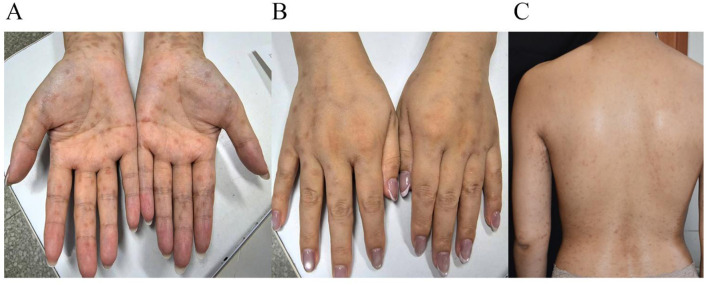
Clinical response after 2 weeks of therapy. **(A)** Marked regression of the lesions on the bilateral palms and volar forearms, with residual post-inflammatory hyperpigmentation. **(B)** Marked improvement of the lesions on the dorsal hands and forearms, with residual hyperpigmented macules. **(C)** Near complete clearance of the lesions on the back, with faint residual post-inflammatory pigmentation.

## 3 Discussion

We present a case of severely pruritic guttate psoriasis that demonstrated an exceptionally rapid and favorable response to oral upadacitinib. Several aspects of this case warrant detailed discussion, including the breakthrough flare during ongoing IL-17A inhibition, the challenging symptom of intractable pruritus, the subsequent role of tonsillectomy in recurrent infection-associated GP, the rationale for choosing a short course of upadacitinib over alternative systemic therapies, and the potential role of JAK1 inhibition as a strategic therapeutic alternative.

First, the occurrence of a widespread GP flare just 1 week after a scheduled dose of secukinumab, a potent IL-17A inhibitor, is noteworthy. Secukinumab is highly effective in both chronic plaque psoriasis and psoriatic arthritis, with emerging evidence in GP (11). The breakthrough flare in our patient suggests that IL-17A-independent inflammatory pathways may contribute to the pathogenesis of acute GP. Indeed, the JAK/STAT pathway integrates signals from multiple upstream cytokines, and its blockade may offer broader suppression of the inflammatory cascade than isolated IL-17A inhibition (15, 22). Thus, upadacitinib may be particularly useful in selected patients who experience incomplete responses or breakthrough flares on biologic monotherapy, although this remains an off-label approach requiring careful patient selection.

Although guttate psoriasis is classically associated with preceding streptococcal infection, the therapeutic value of antibiotics for established guttate psoriasis remains uncertain (23, 24). A Cochrane review of antistreptococcal interventions identified only a small number of randomized trials and concluded that the available evidence was of very low quality and insufficient to determine the efficacy or safety of systemic antibiotics for guttate or chronic plaque psoriasis (23). In particular, a controlled study of streptococcus-associated guttate psoriasis found no statistically significant improvement with oral erythromycin or phenoxymethylpenicillin compared with no treatment (25). Similarly, other clinical studies evaluating penicillin-based regimens, either alone or in combination with other antibiotics, failed to demonstrate consistent additional benefit for psoriasis improvement (26, 27). Therefore, antibiotics should be considered when there is evidence of active streptococcal infection, but an elevated antistreptolysin O titer alone does not necessarily indicate ongoing infection requiring antibiotic therapy (23, 28). In the present case, the absence of clinical and laboratory evidence of active infection supported the decision not to use antibiotics as the primary treatment for the eruption.

Intense pruritus was a prominent feature of the disease in the present case. Although the mechanism of itch in guttate psoriasis has not been fully clarified, psoriatic pruritus is increasingly recognized as a complex neuroimmune symptom involving inflammatory cytokines, neuropeptides, peripheral sensory nerves, transient receptor potential channels, vascular mediators, and epidermal barrier dysfunction (20, 29). Several cytokines implicated in psoriatic inflammation may also contribute to itch. IL-31, a well-recognized pruritogenic cytokine, has been reported to be increased in patients with psoriasis and to correlate with itch severity in some studies (30, 31). In addition, IL-33, TNF-α, IL-17-related inflammation, substance P, and TRP channel activation have been implicated in the amplification of psoriatic itch (20, 29, 32). Importantly, several pruritogenic and inflammatory cytokines, including IL-31, IL-6, IL-22, and interferon-related pathways, signal at least partly through the JAK/STAT pathway (33, 34). Therefore, JAK inhibition may provide dual benefit by suppressing inflammatory signaling and attenuating cytokine-mediated pruritus. This mechanistic rationale is consistent with the rapid relief of pruritus observed in our patient within 24 h after initiation of upadacitinib, before substantial visible improvement of the skin lesions occurred. Nevertheless, because direct evidence for JAK1 inhibition in guttate psoriasis remains limited, this observation should be interpreted cautiously and requires further validation.

Another clinically relevant aspect of this case is the subsequent tonsillectomy. Given the recurrent nature of the patient's GP and its repeated association with upper respiratory infections, tonsillectomy was considered as a potential strategy to reduce future infection-triggered flares. The potential role of tonsillectomy in psoriasis appears to vary across clinical subtypes. It has most commonly been discussed in patients with guttate psoriasis or chronic plaque psoriasis whose disease onset or exacerbations are temporally associated with recurrent tonsillitis or streptococcal pharyngitis (23–25). In a systematic review, Rachakonda et al. reported that among 410 patients with psoriasis who underwent tonsillectomy, 290 experienced clinical improvement; however, most included studies were case reports or case series without control groups, and publication bias should be considered (23). Earlier reports suggested that tonsillectomy may be particularly beneficial in children or young patients with recurrent guttate psoriasis associated with recurrent streptococcal pharyngotonsillitis (24). In addition, a randomized controlled trial in selected patients with chronic plaque psoriasis and a history of sore throat-associated exacerbations showed clinical improvement and improved patient-reported outcomes after tonsillectomy, suggesting that the benefit may not be limited to guttate psoriasis alone (26). Nevertheless, the overall evidence remains insufficient to recommend tonsillectomy as a routine treatment for all patients with psoriasis (27, 28). Therefore, tonsillectomy should be considered only in carefully selected patients, particularly those with recurrent or recalcitrant psoriasis closely linked to tonsillitis or streptococcal throat infection, after multidisciplinary evaluation with otolaryngology. In the present case, the patient had recurrent guttate psoriasis episodes repeatedly associated with upper respiratory infections; therefore, tonsillectomy was considered as an adjunctive strategy to reduce future infection-triggered flares rather than as an acute treatment for the active eruption.

Targeted biologic therapy has been increasingly described in guttate psoriasis (GP), although the available evidence remains limited and is mainly derived from case reports, case series, and small observational studies. Published GP-specific reports have involved several biologic classes, including IL-12/23 inhibition with ustekinumab, IL-23 inhibition with guselkumab, and IL-17 pathway inhibition with ixekizumab, secukinumab, or bimekizumab (11–13, 35–39). Ustekinumab was reported to induce remission in patients with recalcitrant GP (35), while guselkumab has been associated with sustained clearance in several case reports (12, 13, 36). Similarly, IL-17 pathway inhibitors have shown favorable outcomes in selected patients with GP, including durable remission after short-course IL-17 inhibition (37), successful ixekizumab-treated cases (38), and a retrospective cohort of GP patients treated with secukinumab or ixekizumab (11). More recently, short-term bimekizumab therapy has also been reported as a successful approach in recurrent GP (39). Collectively, these reports support the concept that targeted cytokine blockade may be effective in selected cases of severe or refractory GP, but the optimal therapeutic target and treatment duration remain undefined because high-quality GP-specific comparative studies are still lacking.

In the present case, however, the selection of upadacitinib was not based on established evidence for JAK1 inhibition in GP, but rather on the patient's specific clinical context and a mechanistic rationale. The patient developed a severe breakthrough flare only 1 week after scheduled secukinumab administration, suggesting that isolated IL-17A blockade was insufficient to suppress the acute inflammatory cascade in this episode. In addition, the disease was rapidly progressive and accompanied by debilitating, sleep disrupting pruritus that persisted despite conventional symptomatic measures and ongoing biologic therapy. These features made rapid control of both inflammation and pruritus a major therapeutic priority.

The potential therapeutic relevance of JAK/STAT pathway inhibition in psoriasis is supported by accumulating evidence from plaque psoriasis and psoriatic arthritis. Tofacitinib, an oral JAK1/3 inhibitor, demonstrated efficacy in patients with moderate-to-severe plaque psoriasis in phase III trials, and tofacitinib 10 mg twice daily was reported to be non-inferior to etanercept 50 mg twice weekly in one randomized non-inferiority trial (16, 40). Baricitinib, a JAK1/2 inhibitor, also showed significant PASI75 improvement in a phase 2b trial of moderate-to-severe psoriasis (41). More recently, selective TYK2 inhibition has become a major focus in psoriasis therapy. Deucravacitinib, an oral allosteric TYK2 inhibitor, blocks IL-23, IL-12, and type I interferon signaling, thereby targeting pathways central to psoriatic inflammation (42, 43). In the POETYK PSO-1 and PSO-2 phase III trials, deucravacitinib was superior to placebo and apremilast across multiple efficacy endpoints in patients with moderate-to-severe plaque psoriasis (17, 44). Long-term extension studies further demonstrated durable efficacy and a consistent safety profile through several years of treatment (45, 46). In psoriatic arthritis, JAK/TYK2 pathway inhibition has also shown clinical benefit, as demonstrated by studies of tofacitinib, upadacitinib, filgotinib, and deucravacitinib (47–51). These data collectively support the biological plausibility of targeting JAK/STAT-related signaling in psoriatic disease. However, direct evidence for JAK1 inhibition in acute guttate psoriasis remains scarce. Therefore, the use of upadacitinib in the present case should be interpreted as a mechanistically rational but exploratory therapeutic strategy, particularly in the setting of severe, rapidly progressive, and intensely pruritic disease refractory to conventional measures.

Several important safety considerations must be acknowledged. Upper respiratory tract infection, nasopharyngitis, and herpes zoster are recognized infection-related adverse events of JAK inhibitors, including upadacitinib (52–54). This appears clinically paradoxical in GP, a condition often triggered by streptococcal or other upper respiratory infections. In the present case, however, the elevated antistreptolysin O titer was interpreted as evidence of recent exposure rather than active infection, because the patient had no fever, progressive throat symptoms, tonsillar exudate, leukocytosis, elevated inflammatory markers, or other clinical evidence of ongoing infection at treatment initiation. Therefore, the clinical decision was based on the concept that the acute infection had resolved, whereas a post-infectious inflammatory cascade and severe itch remained active. We also minimized risk by using the lowest approved dermatologic dose available to us (15 mg once daily), performing baseline safety evaluation, monitoring closely for infectious symptoms, and discontinuing the drug after 28 days once complete resolution had been achieved. More broadly, upadacitinib also carries risks of serious infections, reactivation of latent tuberculosis, herpes zoster, opportunistic infections, thromboembolic events, and laboratory abnormalities (52–55). Therefore, baseline screening for tuberculosis, viral hepatitis, complete blood count, hepatic and renal function, and careful assessment for active infection are essential before initiation.

The decision to discontinue upadacitinib after 4 weeks was based on the intended role of the drug as a short-term rescue therapy rather than as long-term maintenance treatment. Before treatment initiation, the primary therapeutic goal was rapid control of the acute eruption and debilitating pruritus. By day 28, this target had been achieved, with complete resolution of the skin lesions, a PGA score of 0, a pruritus NRS score of 0, and substantial improvement in quality of life. Therefore, upadacitinib was discontinued after completion of the 28-day course. This decision was not driven by adverse events, laboratory abnormalities, or cost concerns. Instead, it reflected the acute and self-limited nature of the flare, the absence of evidence supporting long-term JAK1 inhibitor maintenance therapy in GP, and the intention to minimize unnecessary immunomodulatory exposure before subsequent elective tonsillectomy. The patient remained free of recurrence during 6 months of follow-up after tonsillectomy, although longer observation is needed to determine whether short-course JAK1 inhibition has any impact on recurrence risk.

In conclusion, this case demonstrates that short-course upadacitinib may represent a valuable therapeutic option for severely pruritic and refractory guttate psoriasis, but only after active infection has been excluded. The rapid onset of action and favorable short-term safety profile observed in this case may provide clinicians with an additional therapeutic option for the management of acute, severe GP, particularly when rapid disease control is needed before subsequent elective interventions such as tonsillectomy. Further studies are required to clarify patient selection, optimal duration, and safety in infection-associated GP.

## Data Availability

The raw data supporting the conclusions of this article will be made available by the authors, without undue reservation.
